# Preserved Expression of Skin Neurotrophic Factors in Advanced Diabetic Neuropathy Does Not Lead to Neural Regeneration despite Pancreas and Kidney Transplantation

**DOI:** 10.1155/2018/2309108

**Published:** 2018-12-10

**Authors:** František Saudek, Monika Cahová, Terezie Havrdová, Klára Zacharovová, Helena Daňková, Luděk Voska, Věra Lánská, Nurcan Üçeyler, Claudia Sommer

**Affiliations:** ^1^Diabetes Center, Institute for Clinical and Experimental Medicine, 14021 Prague, Czech Republic; ^2^Center for Experimental Medicine, Institute for Clinical and Experimental Medicine, 14021 Prague, Czech Republic; ^3^Clinical and Transplant Pathology Department, Institute for Clinical and Experimental Medicine, 14021 Prague, Czech Republic; ^4^Department of Statistics, Institute for Clinical and Experimental Medicine, 14021 Prague, Czech Republic; ^5^University Hospital of Würzburg, Department of Neurology, 97080 Würzburg, Germany

## Abstract

Diabetic peripheral neuropathy (DPN) is a common complication of diabetes with potential severe consequences. Its pathogenesis involves hyperglycemia-linked mechanisms, which may include changes in the expression of neurotrophic growth factors. We analyzed the expression of 29 factors potentially related to nerve degeneration and regeneration in skin biopsies from 13 type 1 diabetic pancreas and kidney recipients with severe DPN including severe depletion of intraepidermal nerve fibers (IENF) in lower limb skin biopsies (group Tx1 1st examination). The investigation was repeated after a median 28-month period of normoglycemia achieved by pancreas transplantation (group Tx1 2nd examination). The same tests were performed in 13 stable normoglycemic pancreas and kidney recipients 6–12 years posttransplantation (group Tx2), in 12 matched healthy controls (group HC), and in 12 type 1 diabetic subjects without severe DPN (group DM). Compared to DM and HC groups, we found a significantly higher (*p* < 0.05–0.001) expression of NGF (nerve growth factor), NGFR (NGF receptor), NTRK1 (neurotrophic receptor tyrosine kinase 1), GDNF (glial cell-derived neurotrophic factor), GFRA1 (GDNF family receptor alpha 1), and GFAP (glial fibrillary acidic protein) in both transplant groups (Tx1 and Tx2). Enhanced expression of these factors was not normalized following the median 28-month period of normoglycemia (Tx1 2nd examination) and negatively correlated with IENF density and with electrophysiological indices of DPN (vibration perception threshold, electromyography, and autonomic tests). In contrast to our expectation, the expression of most of 29 selected factors related to neural regeneration was comparable in subjects with severe peripheral nerve fiber depletion and healthy controls and the expression of six factors was significantly upregulated. These findings may be important for better understanding the pathophysiology of nerve regeneration and for the development of intervention strategies.

## 1. Introduction

Diabetic peripheral neuropathy (DPN) is a chronic complication of diabetes mellitus with potential grave clinical consequences such as pain, loss of sensation, foot ulcers, and gangrene, which may result in amputations. During its natural course, DPN progresses from initial functional to late structural changes with nerve fiber loss at its final stage [1].

Several interrelated pathways linked to chronic hyperglycemia are implicated in the pathogenesis of DPN with oxidative stress playing a major role [2]. There are also experimental and clinical data supporting a contributory role of changes in the expression of vascular and neural growth factors, e.g., vascular endothelial growth factor (VEGF), nerve growth factor (NGF), and other neurotrophins in the pathogenesis of DPN [3].

The principal strategy for the prevention of DPN consists of maintaining long-term optimal glycemic control. However, in spite of major advances in the field, this is still not achievable in a substantial proportion of diabetic patients. Interventions targeting the major putative pathogenic pathways of DPN (antioxidants, aldose-reductase inhibitors, inhibitors of glycation, etc.) have been tested in several randomized controlled trials but had been abandoned due to either toxicity or lack of convincing benefit [4, 5]. Moreover, while being effective as preventive measure, good metabolic control has not been shown so far to reverse already established advanced structural nerve damage and fiber loss in DPN.

Patients with long-standing type 1 diabetes mellitus who undergo pancreas/kidney or pancreas transplantation alone represent a population with very advanced diabetic complications including forms of DPN [6]. Long-term normoglycemia can be reestablished after successful pancreas transplantation without further need of insulin injections. Longitudinal follow-up of type 1 diabetic patients undergoing a pancreas (and kidney) transplantation may thus provide novel evidence on the effect of long-term normoglycemia in advanced forms of DPN.

Past trials of preventive and therapeutic interventions in DPN have mostly used clinical symptoms and signs and results of quantitative sensory testing or electrophysiological studies as outcome measures. The introduction of the minimally invasive technique of skin biopsy with determination of intraepidermal nerve fiber (IENF) morphology provides an opportunity for the quantitative assessment of the treatment [7]. In addition, skin biopsy samples can be analyzed for changes in neurotrophic factors in relation to the pathophysiology of neuropathies including diabetic neuropathy [8, 9]. A decreased nerve regenerative capacity has been associated with impaired neurotrophic tone which might reflect to diminished synthesis, secretion, or action of neurotrophic factors in sensory and autonomic nerve fibers [9]. Besides well-established trophic factors, such as the members of the neurotrophin family like NGF or VEGF, novel neurotrophic factors are being discovered which may be deficient in patients with diabetes. Furthermore, the signal transduction pathways of neurotrophic factors may be changed in diabetes and may contribute to either disrupting neuronal survival or nerve regeneration [10].

In our prospective studies which included type 1 diabetic subjects with advanced peripheral neuropathy, we showed that IENF density did not improve even after 2.5 and 8 years of normoglycemia after successful pancreas and kidney transplantation [11, 12]. It is still unknown whether long-term improvement of glycemic control may alter skin neurotrophin levels in advanced neuropathy. We hypothesized that expression of selected neurotrophins and growth factors in the skin might correlate with clinical signs of diabetic peripheral neuropathy and, in contrast to unchanged epidermal nerve fiber density, might be altered following the reestablishment of normoglycemia after pancreas transplantation as a tissue stimulus for neural regeneration. This could provide novel evidence in relation to nerve regeneration following the reestablishment of normal glucose levels.

## 2. Methods

### 2.1. Study Design

A comprehensive neurological evaluation comprising clinical assessment, electromyography, autonomic tests, and skin biopsy for IENF quantification and measurement of mRNA of selected neurotrophic factors and receptors was performed in three groups of type 1 diabetic subjects (groups DM, Tx1, and Tx2) and in nondiabetic age- and sex-matched healthy controls (HC). The study was performed at the Institute for Clinical and Experimental Medicine (IKEM) in Prague in 2012–2015 and was approved by the Ethics Committee of IKEM and Thomayer's Teaching Hospital with registration no. G-12-06-43. After the explanation of the aims and study procedures, all participants signed an informed consent to participate in the study.

The DM group included 12 subjects with type 1 diabetes without clinical symptoms of diabetic neuropathy according to the diabetic neuropathy symptom score [13] and a VPT below 15 V. Group Tx1 included 13 subjects who were examined within 2 and 20 weeks (median 8 weeks) following successful pancreas and kidney transplantation (Tx1 1st examination) and reevaluated again within 15 to 30 months (median 28 months; Tx2 2nd examination) of normal function of both grafts. Group Tx2 was comprised of 13 type 1 diabetic subjects who had been normoglycemic for at least eight years after successful pancreas and kidney transplantation. Demographic data for individual groups are shown in Table 1.

#### 2.1.1. Transplantation

Groups Tx1 and Tx2 comprised patients with advanced complications of diabetes including severe diabetic neuropathy and end-stage diabetic nephropathy. Severe diabetic neuropathy was defined by an experienced neurologist by evaluating the neuropathy symptom score, VPT, and EMG. All patients underwent successful pancreas and kidney transplantation and were off exogenous insulin and normoglycemic and had good kidney graft function at the time of study evaluation. The kidney graft was placed into the left iliac fossa. The pancreatic graft was placed intraperitoneally either with systemic or portal venous drainage and anastomosis of the donor duodenum segment to the intestine. Immunosuppressive therapy is comprised of 4-dose induction with anti-T-lymphocyte globulin (Fresenius Biotech GmbH, Gräfelfing, Germany) and prophylactic combination of tacrolimus (through levels 8–15 ng/ml) with either mycophenolate mofetil (1–2 g per day) or sirolimus (trough levels 5–10 ng/ml). Corticosteroids were started at a dose of 20 mg per day and gradually decreased and discontinued 6 weeks after transplantation.

Subjects in both the Tx1 and the Tx2 groups were enrolled into the study if they fulfilled the following criteria: severe neuropathy before transplantation, no major complication and a rejection-free course after transplantation with normoglycemia, good function of the kidney graft, and informed consent to participate in the study.

#### 2.1.2. Skin Biopsies and IENF Density

Skin biopsies were taken under local anesthesia from the thigh (10–15 cm above the knee) using a 3 mm punch (Stiefel Laboratories, Sligo, Ireland). No sutures were needed due to the biopsy size. Three samples (1 for ENF counts and 2 for mRNA analyses) were taken from each subject.

Samples for IENF density determination were fixed in 4% paraformaldehyde and then processed as previously described [11, 12]. For fluorescence imaging of the panaxonal marker protein gene product 9.5 (DakoCytomation, Glostrup, Denmark), 3 sections per patient were examined. The mean number of IENF per millimeter of epidermis was derived using the software Olympus DP-SOFT (Software Imaging Systems, Münster, Germany).

For RT-qPCR analysis, the skin samples were immediately immersed into RNAlater (Sigma), left for 2 h at 4°C, and then stored at −80°C until analysis.

#### 2.1.3. Electromyography

Nerve conduction studies were carried out using standard techniques on the 3-channel Keypoint Dantec Focus device. Motor nerve conduction velocity (NCV) and a compound muscle action potential of the ulnar and tibial nerve were measured orthodromically. Sensory NCV and amplitude of sensory nerve action potential were measured antidromically in the ulnar and sural nerve. Skin temperature was approximately constant (32°C) during the examinations in both upper and lower extremities.

#### 2.1.4. Vibration Perception Threshold (VPT)

The VPT was examined using the biothesiometer probe (Bio-Thesiometer; Bio-Medical Instruments, Newbury, OH) placed over the dorsum of the hallux.

#### 2.1.5. Autonomic Tests

Heart rate responses to deep breathing and the Valsalva maneuver and heart rate and systolic blood pressure responses to standing up, belonging to a battery of standard autonomic function tests (AFTs), were performed with Varia Pulse TF3, a telemetric online computer-aided system (Sima Media Ltd., Olomouc, Czech Republic). Blood pressure was measured with an automated oscillometric device (Omron M4; Matsusaka, Japan). The main outcome parameters were the I-E difference (mean difference of maximal inspiratory and minimal expiratory heart rate), Valsalva ratio (VR; maximal to minimal heart rate during and following the Valsalva maneuver), 30 : 15 ratio (ratio of heart rates around the 15th and 30th heart beats following standing up), and Δ sBP (difference in systolic blood pressure while supine and at 1 min after standing up).

#### 2.1.6. RNA Extraction, Reverse Transcription PCR, and Gene Expression Analysis

After thawing, skin samples were immersed in 1 ml TRIzol reagent (Sigma), dispersed by knife homogenizer (IKA Ultra Turrax, Germany), and extracted into chloroform. RNA isolation from the chloroform extract was performed using RNeasy Mini Kit (Qiagen) according to the manufacturer's instructions. All PCR reagents and cyclers were purchased from Thermo Fisher Scientific (Foster City, CA). Extracted mRNA (300 ng) was reverse transcribed using SuperScript VILO (Invitrogen). The expression of selected genes was determined using custom-designed TaqMan Gene Expression Array Cards with the following gene-specific TaqMan Assays: CNTF (Hs04194755_s1), CNTFR (Hs00181798_m1), GALR1 (Hs00175668_m1), GALR2 (Hs00605839_m1), GFRA1 (Hs00237133_m1), GFRA2 (Hs00176393_m1), GFRA3 (Hs00181751_m1), NGF (Hs00171458_m1), NGFR (Hs00609977_m1), NGFRAP1 (Hs00918411_s1), NRG1 (Hs00247620_m1), NRG2 (Hs00171706_m1), NRG4 (Hs00945535_m1), NTF3 (Hs00267375_s1), NTF4 (Hs01596132_m1), NTRK1 (Hs01021011_m1), NTRK2 (Hs00178811_m1), TGFA (Hs00608187_m1), NPY (Hs00173470_m1), NPYR1 (Hs00702150_s1), BDNF (Hs03805848_m1), MPZ (Hs00559329_m1), GFAP (Hs00909233_m1), FGF2 (Hs00266645_m1), FGFR1 (Hs00915142_m1), GDNF (Hs01931883_s1), ERBB2 (Hs01001580_m1), ERBB3 (Hs00176538_m1), and ERBB4 (Hs00955525_m1) (Thermo Fisher Scientific). The suitable endogenous controls, HPRT 1 (Hs99999909_m1) and B2M (Hs99999907_m1), were selected by TaqMan Array Human Endogenous Control Panel (cat. no.: 4366071) as the genes with most stable expression. The analyses were performed on ABI 7900HT Sequence Detection System. Samples were measured as triplicates. All plates were analyzed applying identical conditions. We used the comparative ∆Ct method (i.e., relating target gene expression with individual HPRT and B2M expression) for individual assessment. In addition, we compared gene expression in the patient and control groups using the ∆∆Ct method when the average normalized expression of each target gene in the skin samples from the healthy control group was used as calibrator. The list of examined neurotrophic factors and their receptors is given in Table 2.

### 2.2. Statistical Evaluation

All data are expressed as median and range (tables) or median and 1st/3rd quartile plus outliers (graphs). The differences among the groups were first compared using the Kruskal-Wallis analysis of variance, and if *p* values < 0.05 were found, the differences between individual groups were compared using the Wilcoxon test with Bonferroni correction. Paired data (group Tx2, the 1st and the 2nd examination) were tested by the Wilcoxon signed-rank test. The data obtained by RTq-PCR arrays were analyzed using RT^2^ Profiler™ PCR Array Data Analysis software provided by the company together with the PCR array. The relationship between individual parameters was assessed using the Spearman's rank correlations test. Correlations with a *p* value < 0.05 were considered as significant.

## 3. Results

### 3.1. Demography and Metabolism

There were no differences among the 4 groups of subjects in terms of age and BMI (*p* > 0.05, Table 1). Both transplant groups before transplantation and diabetic control subjects (group DM) had elevated values of HbA1c. At the time of examination, however, the HbA1c values in both transplant groups did not differ from healthy subjects, although in the Tx1 group some values at the 1st examination were still abnormal, as the time from transplantation was too short for normalization. High serum creatinine levels in both transplant groups rapidly dropped following the combined pancreas and kidney transplantation and remained stable during the whole follow-up period with the highest registered level at the time of study examination being 186 *μ*mol/l (see Table 1). None of the transplant recipients needed insulin or oral antidiabetic therapy during the study follow-up.

### 3.2. Vibration Perception Threshold (VPT)

Median VPT values in groups HC, DM, Tx1, and Tx2 were 10, 13, 34, and 30, respectively, and were significantly higher in both transplant groups than in the HC and DM groups (*p* < 0.001 and <0.05, respectively). The difference between the diabetic and healthy control groups was not significant. In the Tx1 group, the VPT did not improve during the study follow-up period with the median values remaining almost the same.

### 3.3. Electromyography

Electrophysiological data are presented in Table 3.

After Bonferroni correction, most nerve conduction velocities and amplitudes were significantly more pathological in the Tx1 and Tx2 groups compared to healthy controls (*p* < 0.05, *p* < 0.001). In the DM group, only ulnar sensory NCV was significantly lower than in healthy controls (*p* = 0.034). Tibial NCV (*p* = 0.0625) showed borderline significance for improvement between the 1st and 2nd examinations in group Tx1.

### 3.4. Autonomic Tests

The results are summarized in Table 4. Parameters I-E difference and Δ sBP in both transplant groups were significantly more pathological than in both control groups (*p* < 0.05 and *p* < 0.01, respectively). Valsalva ratio was lower in the Tx1 and Tx2 groups than in the HC group (*p* < 0.05 and *p* < 0.01, respectively). Both transplant groups vs. HC group and Tx1 vs. DM groups significantly differed in 30 : 15 ratio (*p* < 0.05).

### 3.5. Intraepidermal Nerve Fiber Density

**Figure 1:**
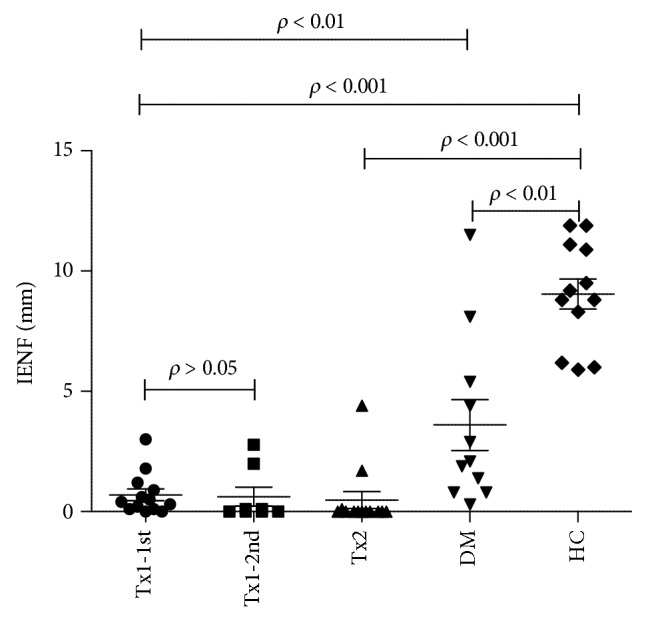
Scatterplot, IENF-density.

In both transplant groups, no or very few IENF were found in skin biopsy samples. Median and range values in the Tx1 and Tx2 groups were 0.4 (0–3) and 0 (0–4.41), respectively, and were significantly lower than in the HC (8.3 (5.9–11.1); *p* < 0.01) or DM (2.1 (0.3–11.5); *p* < 0.05) controls. There was no improvement in group Tx1 after the median 28-month period of normoglycemia (*p* = 0.17). Graphical comparison of the IENF densities is shown in Figure 1.

### 3.6. Expression of Neurotrophic Factor mRNA in Skin Biopsies

**Figure 2:**
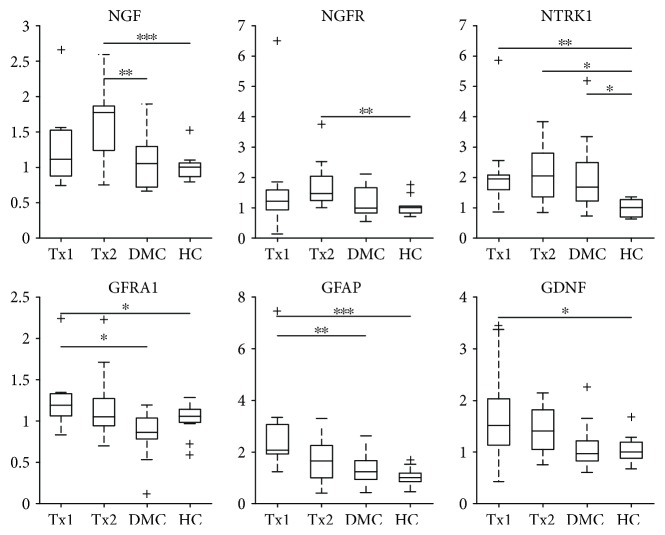
Relative expression of six differently expressed factors. Data are shown as median and 1st and 3rd quartile. Differences between individual groups were assessed by Wilcoxon test with Bonferroni correction: ^∗^ *p* < 0.05; ^∗∗^ *p* < 0.01; ^∗∗∗^ *p* < 0.001. NGF: nerve growth factor, NGFR: NGF receptor, NTRK1: neurotrophic receptor tyrosine kinase 1, GDNF: glial cell-derived neurotrophic factor, GFRA1: GDNF family receptor alpha 1, GFAP: glial fibrillary acidic protein. +: outlying values.

The expression of 29 neurotrophic and related factors in skin samples was compared among the four study groups using the Wilcoxon/Kruskal-Wallis tests. We found significant differences in six: NGF (*p* < 0.001), NGFR (*p* < 0.05), NTRK1 (*p* < 0.01), GFRA1 (*p* < 0.01), GFAP (*p* < 0.001), and GDNF (*p* < 0.05) (see Figure 2). In addition, we found marginal differences (*p* < 0.08) in the expression of NRG2, TGFA, and FGF2. Interestingly, the expression of all these nine factors was higher in both transplant groups (Tx1 and Tx2) than in both HC and DM controls (Supplemental Table 1).

**Figure 3:**
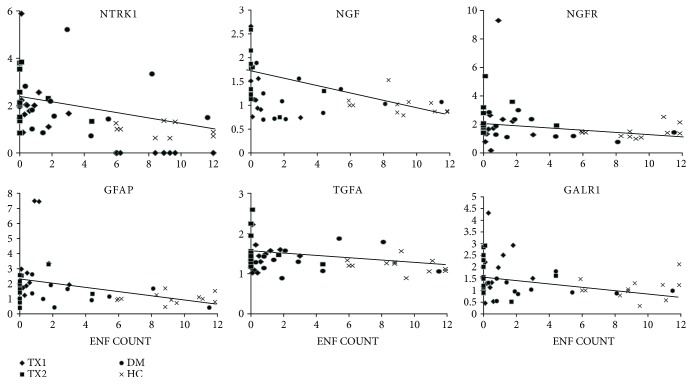
Correlation of intraepidermal nerve fiber density and expression of selected neurotropic factors. IENF count (*x*-axis) is expressed as number of fibers/mm; relative expression of selected neurotrophins (*y*-axis) is calculated by ΔΔCt method using normalized expression of each target gene in the HC group as a calibrator.

For the six parameters that had a higher expression in groups Tx1 and Tx2 compared to HC and DM, we examined their correlation with the IENF density assessed by fluorescence microscopy (Figure 3). This correlation was statistically significant in all cases with the *p* value being <0.001 with GFR, <0.01 with NTRK1 and GFAP, and <0.05 with NGFR, GALR1, and TGFA.

**Figure 4:**
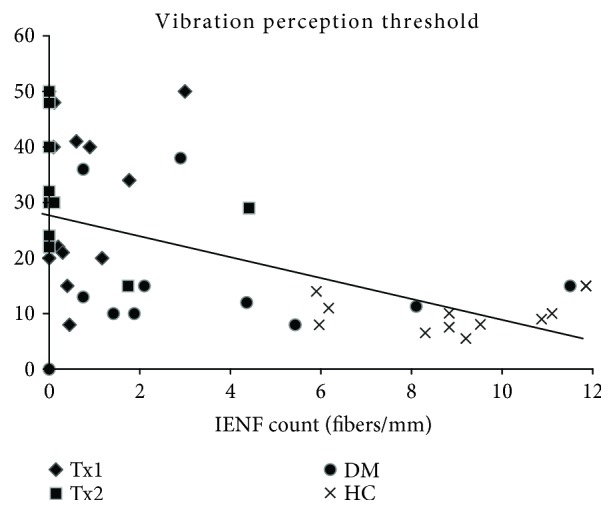
Correlation of intraepidermal nerve fiber density (IENFD) and vibration perception threshold.

We also found a significant correlation between IENF density and clinical indicators of neuropathy severity. As an example, the correlation between IENF density and vibration perception threshold is shown in Figure 4 (*r* = −0.63, *p* < 0.001).

Significantly negative correlations were found between the parameters of autonomic neuropathy, nerve conduction velocities/amplitudes, and expression of selected factors. In the case of vibration perception threshold and amplitudes, the correlations were positive. These significant correlation were mainly the same neurotrophic factors as per the IENF density, i.e., GALR1, NGF, NGFR, NTRK1, TGFA, and GFAP. This finding supports the hypotheses that enhanced expression of neurotropic factors is associated with the severity of the neuropathy.

Using stepwise multiple linear regression comprising these 6 factors, we found that IENF density was mainly dependent on GFAP (*p* = 0.014) and NGF (*p* = 0.035). These two parameters accounted for 29% of the IENF density variance.

**Figure 5:**
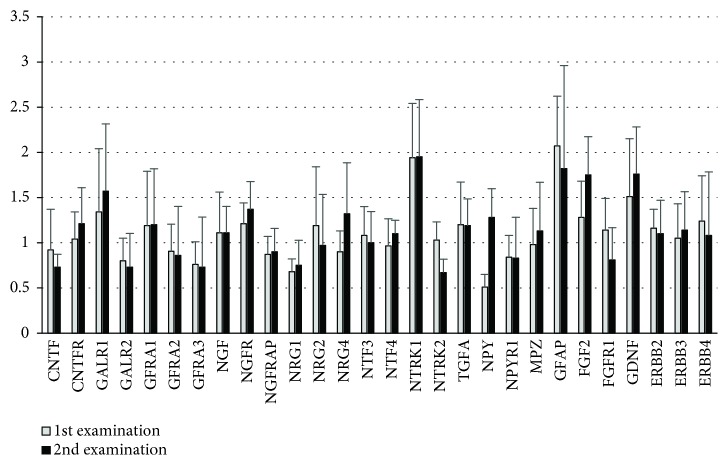
Comparison of the neurotropic factor expression profile in skin biopsies in Tx1 1st and 2nd examinations. The 1st examination was performed early after transplantation and the 2nd examination after the median 28-month period of normoglycemia. Data shown as median and 3rd quartile values. Wilcoxon signed-rank test: *p* > 0.05 for all parameters. The *y*-axis shows a relative expression of selected neurotrophins calculated by ΔΔCt method using a normalized expression of each target gene in HC group as a calibrator.

The neurotropic expression profiles in group Tx1 assessed early after transplantation and after the median 28-month period of normal glucose induced by pancreas transplantation did not change significantly (Figure 5).

## 4. Discussion

We have investigated skin expression of selected neurotrophic factors and their receptors in subjects with advanced peripheral diabetic neuropathy. In agreement with the experimental data and prevailing clinical views [9, 18, 19, 30], we expected lower expression that would correspond to tissue injury caused by longstanding hyperglycemia and multifaceted metabolic disorders. This assumption, however, was not confirmed. In contrast, with one exception (see Supplemental Table), the expression of all factors was either equal or increased in the Tx1 and Tx2 groups compared with both healthy subjects and diabetic patients without clinically evident neuropathy. Although the upregulated gene expression may not necessarily imply an escalation of protein transcription, we consider this finding novel and important in the search for new targets to stimulate neural regeneration in advanced diabetic neuropathy. Of note, some of these factors had been assessed previously as they could possibly ameliorate advanced nerve damage or they could be of interest for possible interventions [18, 31].

We also conclude that the expression of neurotrophic factors did not change after restoration of endogenous insulin secretion and normalization of glucose homeostasis through pancreas transplantation after 28 months or 8 years of long-lasting normoglycemia when compared with healthy subjects and diabetic subjects without clinically evident neuropathy. At the same time, the enhanced expression profile did not result in any demonstrable regeneration of peripheral sensory nerve fibers. Skin levels of neurotrophins have been assessed in very few studies with somewhat contradictory results, with depletion of skin NGF in patients with diabetic neuropathy [9], and also increased NGF mRNA [17], neurotrophin-3 (NTF3) concentration [32], and expression of the trkA and trkC receptors for NGF and NTF3, respectively [33]. The situation is further complicated by the effect of the duration of diabetes. In an experimental study, Terada et al. [34] demonstrated that motor nerve regeneration after partial denervation was decreased in long-term diabetic rats, whereas it was enhanced in short-term diabetic animals. In a recent work, Uceyler et al. demonstrated decreased skin expression of NGF, NT3, and erythropoietin genes in chronic neuropathies, but the study included only 4 diabetic subjects [8].

A complex analysis of the expression profiles in sural nerve biopsies was performed in 36 subjects with mild to moderate neuropathy in predominantly type 2 diabetic subjects as a part of a larger intervention study [35]. The bioinformatics analysis identified a group of genes which were differentially expressed in samples from patients who showed neuropathy progression over a 52-week period or not. Subsequent examination revealed their relation mainly to inflammatory and lipoprotein subnetworks rather than neurotrophic factors and their receptors. However, this study did not include a nondiabetic control group and assessed progression rather than severity of neuropathy. Further expression studies in skin samples (which are much easier to perform than sural nerve biopsies) should include the candidate genes which have been identified in this work.

Despite the potential effects of the concomitant immunosuppressive therapy, pancreas transplantation represents an optimal model to study the effects of reestablishment of stable normoglycemia in advanced diabetic neuropathy and provides an opportunity to examine whether correction of the principal metabolic noxa, hyperglycemia, may lead to any functional and morphological improvement. In a retrospective analysis of changes in myelinated fiber density in sural nerve biopsies over a 52-week period in mostly type 2 diabetic subjects, HbA1c levels significantly correlated with density improvement but patients with only mild to moderate neuropathy were included [36].

In pancreas and kidney recipients, mostly with advanced neuropathy, several previous studies demonstrated a significant regression of neuropathic symptoms and improvement of electrophysiological indices [37–39]. However, the regenerative capacity probably depends on the stage of the disease [40]. In subjects with impaired glucose tolerance and mild neurological disease, a diet and exercise program was associated with significant increases in intraepidermal nerve fiber density related to improvements in pain and sural nerve action-potential amplitude [41]. In a small study in DPN with the anticonvulsive topiramate, Boyd et al. reported improvements in epidermal nerve fiber density and length [42]. In our recent 8-year prospective work, with the exception of a single patient, we did not show regeneration of epidermal nerve fibers in a group of 12 normoglycemic pancreas and kidney recipients with advanced neuropathy [12]. More optimistic results have been reported with the use of corneal confocal microscopy showing improved density and length of corneal nerve fibers 12 months after SPK [43]. However, the effect in corneal nerves may not predict meaningful neural regeneration elsewhere in the body [44].

We consider it important to know if there is any local attempt to repair severe neural damage. A deficient local response could reflect the consequence of lengthy metabolic disorder. Despite this, we showed that the expression especially of NGF, NGFR, NTRK1, GFRA1, GFAP, and GDNF was upregulated in patients with severe neuropathy as compared to healthy subjects and diabetic patients with subclinical neuropathy. This upregulation of neurotropic factors, however, did not lead to an improvement of electrophysiological parameters or ENF density despite long-term blood glucose normalization and persistence of the enhanced expression profile at 2 and 8 years.

An important shortcoming of our study is that we could not detect the abundance of the specific proteins as we did not have sufficient material for either immunofluorescent or Western blot analysis. We also did not measure the expression of VEGF, which may contribute to the development of neuropathy through its effect on nerve microcirculation or through a direct effect on neurons and other cellular peripheral nerve components. While a decreased production of NGF in various tissues has been implicated in the pathogenesis of experimental diabetic polyneuropathy [45–47], in animal studies, impaired retrograde axonal transport of NGF is also partly responsible for the reduced nerve regenerative capacity [48]. Its increased expression in skin tissue is thus at odds with these findings. Another possibility is that these protein factors need not be transcribed in fully functional forms [19] or, for their full engagement, additional changes are required. We also were not able to determine the specific origin of the upregulated mRNAs which might come from skin fibroblasts, endothelial, or Schwann cells. The most important conclusion of our study is that local reparatory stimuli in severely nerve-depleted tissue are present but are not efficient.

We acknowledge the lack of a group of patients with advanced diabetic peripheral neuropathy represents a considerable limitation of our study. The local transcription profile of neurotrophic factors at 2–20 (median 8) weeks following successful pancreas and kidney transplantation may not sufficiently reflect the situation before transplantation. Nevertheless, routine performance of skin biopsies in transplant candidates with end-stage kidney failure would have been problematic not only due to a higher risk of local complications. Additionally, waiting times differ among subjects and not all of them reach normal blood glucose levels and good kidney function without episodes of rejection.

This study provides further support to the idea that any therapeutic intervention for diabetic neuropathy should be initiated in the earlier stages before severe neural depletion. Improved metabolic control may be achieved using qualified patient education and emerging technological strategies such as continuous measurement of interstitial glucose level and semiautomatic use of insulin pumps. But if these therapeutic measures fail, then whole organ pancreas or isolated islet transplantation should be considered in subjects with poor metabolic control who do not suffer from advanced diabetic neuropathy.

It remains questionable, whether nerve fiber regeneration in such advanced stages is possible. Stimulating factors may arise from the proximal axons and are transported distally. However, in the case of severe axon damage or death of the whole nerve, regeneration may not by possible unless new neural cells can be transplanted or developed from other cell types.

## Figures and Tables

**Table 1:** Background demographic data and clinical variables.

		Group HC: healthy control subjects	Group DM: type 1 diabetic subjects	Group Tx1: SPK recipients reexamined at 14–30 months	Tx2: SPK recipients at 8 years following transplantation
*n*		12	12	13	13
Age (years)		43 (28–56)	37 (32–55)	39 (30–57)	44 (33–59)
BMI (kg/m^2^)		26.5 (22–37)	26 (22–36)	22.8 (19–28)	24 (20–29)
Diabetes duration (years)		—	18 (4–36)	22 (16–39)	23 (19–50)
Insulin dose (IU/day)		—	48.5 (40–71)	—	—
Pretransplant insulin dose		—	—	32 (20–61)	35 (16–54)
HbA1c	mmol/mol %	36 (25–37) 5.4 (4.4-5.5)	57.5 (47–79) 7.4 (6.4–9.4)	40 (34–52)/40 (35–54) 5.8 (5.3–6.9)/5.8 (5.3–7.1)	37 (32–43) 5.5 (5.1–6.1)
Pretransplant HbA1c	mmol/mol %	—	—	74 (45–99) 8.9 (6.3–11.2)	63 (44–84) 7.9 (6.2–9.8)
Serum creatinine (*μ*mol/l)		81.3 (59–112)	86.1 (56–101)	103 (62–186)/103.7 (81–154)	100 (75–121)

Data are given as medians and range (in parentheses). If not stated otherwise, the values were determined at the time of investigation.

**Table 2:** List of examined neurotrophic factors and their receptors.

Abbrev.	Name	NCBI reference sequence	Potential role in nerve regeneration	Reference
BDNF	Brain-derived neurotrophic factor	NM_001709	Promotion of nerve cell growth and maturation	[14]
CNTF	Ciliary neurotrophic factor	NG_008776.1	Expressed in myelinating Schwann cells, nerve regeneration following injury	[10]
CNTFR	Ciliary neurotrophic factor receptor	NG_047044.1		[10, 15]
GALR1	Galanin receptor 1	NG_009223.1	Neuroprotection, neuroregeneration	
GALR2	Galanin receptor 2		Neuroprotection, neuroregeneration Expressed in Schwann cells, ensures the response to GDNF	[15, 16] (2002)
GFRA1	GDNF family receptor alpha 1	NG_050620.1		
GFRA2	GDNF family receptor alpha 2	NG_029215.1	Expressed in Schwann cells, ensures the response to GDNF Essential role in neuronal regeneration	[16–18, 19]
GFRA3	GDNF family receptor alpha 3	NG_046894.1		
NGF	Nerve growth factor	NG_007944.1		
NGFR	Nerve growth factor receptor	NC_000017.11	Essential role in neuronal regeneration	[19, 20]
NGFRAP	Nerve growth factor receptor associated protein 1	NM_0012826741	Essential role in neuronal regeneration	[19]
NRG1	Neuregulin 1	NG_012005.1	Family of gliotrophic factors that transduce signal through ErbB receptors and are necessary for Schwann cells growth, survival, and differentiation	[21]
NRG2	Neuregulin 2	NC_000005.10	Family of gliotrophic factors that transduce signal through ErbB receptors and are necessary for Schwann cells growth, survival, and differentiation Increase of NT-3 appears to reflect the degree of skin denervation in diabetic neuropathy and may represent a compensatory mechanism	[10, 21]
NRG4	Neuregulin 4	NC_000015.10		
NTF3	Neurotrophin 3	NG_050629.1		
NTF4	Neurotrophin 4	NG_016289.1	Increase of NT-3 appears to reflect the degree of skin denervation in diabetic neuropathy and may represent a compensatory mechanism Essential role in neuronal regeneration	[10, 21, 22]
NTRK1	Neurotrophic receptor tyrosine kinase 1	NG_007493.1		
NTRK2	Neurotrophic receptor tyrosine kinase 2	NG_012201.2	Essential role in neuronal regeneration	[21, 22]
TGFA	Transforming growth factor alpha	NG_029975.1	Trophic factor in both central and peripheral neural tissues	[23]
NPY	Neuropeptide Y	NG_016148.1	Increased expression in patients with moderate sensory neuropathy	[24]
NPY1R	Neuropeptide Y receptor Y1	NC_000004.12	Potentially involved in neuronal precursor proliferation	
MPZ	Myelin protein zero	NG_008055.1	Myelin-specific protein, Schwann cells can modulate this gene expression in response to diabetic-induced metabolic derangement	[25]
GFAP	Glial fibrillary acidic protein	NG_008401.1	Glial cell marker	[26]
FGF2	Fibroblast growth factor 2	NG_029067.1	Expressed by the Schwann cells of the peripheral nerves	[27]
FGFR1	Fibroblast growth factor receptor 1	NG_007729.1	Secreted by Schwann cells, essential for neuroregeneration	[16, 27, 28]
GDNF	Glial cell-derived neurotrophic factor	NG_011675.2	Rapidly responses to denervation produced by basal keratinocytes and Schwann cells	
ERBB2	erb-b2 receptor tyrosine kinase 2	NG_007503.1	Neuregulin receptors, response to chronic denervation, expressed in Schwann cells (26 hoke)	[21, 29]
ERBB3	erb-b2 receptor tyrosine kinase 3	NG_011529.1	Neuregulin receptors, response to chronic denervation, expressed in Schwann cells	[21, 29]
ERBB4	erb-b2 receptor tyrosine kinase 4	NG_011805.1		

**Table 3:** Electrophysiological data.

	Group	HC	DM	Tx1		Tx2
				1st exam.	2nd exam.	
Ulnar nerve sensory	NCV (m/s)	55.9 (50.6–31.3)	50.8 (42–56.6)^∗^	37.1 (20.3–62.2)^∗^	32.1 (20.4–58.4)	47 (37.1–52.4)
	Amp (*μ*V)	20.7 (12.8–41.7)	15.4 (0.9–31.5)	5.8 (−0.3–18.5) ^∗∗^	10.7 (4.1–35.5)	5.8 (2.9–12.1)^∗∗∗^

Sural nerve sensory	NCV (m/s)	54.9 (23.8–64.2)	42.7 (21.3–60.1)	38.5 (22.6–66.4)	48.9 (37.5–64.8)	33.8 (27.5–41.1)
	Amp (*μ*V)	10.7 (5.3–18.2)	8.3 (3.5–17.9)	6 (2–18)	5.9 (3.5–7)	6 (5.2–6.2)

Ulnar nerve motor	NCV (m/s)	61 (51–72)	54.8 (48–62.7)	50.4 (46.8–58.9)^∗∗^	52.2 (42.7–56.4)	55.2 (51.4–57.2)
	Amp (mV)	7.5 (5.7–10.8)	7.5 (5.4–9.9)	4 (1.4–7.8)^∗∗^/xx	4.4 (2.4–7.1)	6 (36.4–43.4)

Tibial nerve motor	NCV (m/s)	49.5 (43.3–53.2)	43.6 (37.4–50.6)	37.4 (33.5–44.7)^∗∗^	43.8 (36–47.5)	41.5 (36.4–43.4)^∗∗^
	Amp (mV)	9.4 (5–18.6)	7.9 (2.5–17.1)	3 (0.2–0.5)^∗^	5.5 (1–11.4)	3.1 (0.8–3.8)^∗∗^/x

Data are given as medians and range (in parentheses). ^∗^ versus HC *p* < 0.05; ^∗∗^ versus HC *p* < 0.01; ^∗∗∗^ versus HC *p* < 0.001; x versus DM *p* < 0.05; xx versus DM *p* < 0.01.

**Table 4:** Results of autonomic tests.

	Group	HC	DM	Tx1		Tx2
				1st exam.	2nd exam.	
I-E difference	(beats/min)	18.8 (8.1–33.9)	20.4 (6.4–29.3)	3.1 (0.9–9.9)^∗∗∗^/xxx	4.4 (0.8–14.9)	4.9 (2.9–7.8)^∗∗^/x
Valsalva ratio		1.7 (1.3–2.1)	1.7 (1.1–2.6)	1.2 (1.1–1.6)^∗^	1.3 (1.1–1.6)	1.2 (1.1–1.2)^∗∗^
30 : 15 ratio		1.13 (0.9–1.14)	1.12 (0.9–1.4)	0.98 (0.9–1.1)^∗^/x	0.99 (0.9–1.1)	0.97 (0.96–1)^∗^
Δ sBP	(mmHg)	1 (−14–12)	3 (−10–17)	37 (2–69)^∗∗^/xx	18 (4–66)	40 (26–59)^∗∗^/xx

Data are given as medians and range (in parentheses). ^∗^ versus HC *p* < 0.05; ^∗∗^ versus HC *p* < 0.01; ^∗∗∗^ versus HC *p* < 0.001; x versus DM *p* < 0.05; xx versus DM *p* < 0.01.

## Data Availability

All data used to support the findings of this study are available from the corresponding author upon request. Specifically, primary data concerning all clinical assessments are stored by FS and TH, histological data by LV, and expression studies data by MC from the authors' team. Additional information concerning the study results is given in Supplementary Tables 1, 2, and 3.

## Abbreviations

AFT: Autonomic function test
Amp: Amplitude
DPN: Diabetic peripheral neuropathy
HPRT: Hypoxanthine phosphoribosyl transferase
IENF: Intraepidermal nerve fibers
I-E difference: Mean difference of maximal inspiratory and minimal expiratory heart rate
NCV: Nerve conduction velocity
SPK: Simultaneous pancreas and kidney transplantation
VEGF: Vascular endothelial growth factor
VPT: Vibration perception threshold
VR (Valsalva ratio): Maximal to minimal heart rate during and following the Valsalva maneuver
30 : 15 ratio: Ratio of heart rates around the 15th and 30th heart beats following standing up
Δ Ct: Difference in Ct_q_ values
Δ sBP: Difference in systolic blood pressure while supine and at 1 min after standing up.

## Supplementary Materials

Supplementary Materials Supplemental Table 1: expression of individual neurotrophic factors mRNA in skin biopsies (median, minimal, and maximal values) in individual groups. Supplemental Table 2: Spearman's rank correlation—intraepidermal nerve fiber (IENF) count versus individual factors. Supplemental Table 3: Spearman's rank correlation—vibration perception threshold assessed by biothesiometer (BTM1) versus individual factors.
