# Supplementary material for: Preserved Expression of Skin Neurotrophic Factors in Advanced Diabetic Neuropathy Does Not Lead to Neural Regeneration despite Pancreas and Kidney Transplantation
Source: J Diabetes Res. 2018 Dec 10;2018:2309108. doi: 10.1155/2018/2309108 (PMC6311823; doi:10.1155/2018/2309108)
Supplement: Supplementary Materials — Supplemental Table 1: expression of individual neurotrophic factors mRNA in skin biopsies (median, minimal, and maximal values) in individual groups. Supplemental Table 2: Spearman's rank correlation—intraepidermal nerve fiber (IENF) count versus individual factors. Supplemental Table 3: Spearman's rank correlation—vibration perception threshold assessed by biothesiometer (BTM1) versus individual factors. [file 2309108.f1.docx]

Supplemental table 1

Individual factors (median. minimal and maximal values) in individual groups

|  | **Tx1** | | | **Tx2** | | | **DM** | | | **HC** | | |
| --- | --- | --- | --- | --- | --- | --- | --- | --- | --- | --- | --- | --- |
|  | median | min | max | median | min | max | median | min | max | median | min | max |
| **CNTF** | 0.9 | 0.8 | 1.7 | 0.9 | 0.8 | 1.6 | 0.9 | 0.7 | 1.4 | 1.0 | 0.7 | 1.4 |
| **CNTFR** | 1.0 | 0.5 | 4.5 | 1.1 | 0.5 | 3.0 | 0.9 | 0.3 | 1.3 | 1.0 | 0.7 | 1.5 |
| **GALR1** | 1.3 | 0.5 | 4.3 | 1.6 | 0.5 | 2.9 | 1.0 | 0.6 | 1.8 | 1.0 | 0.3 | 2.1 |
| **GALR2** | 0.8 | 0.5 | 2.5 | 1.2 | 0.7 | 1.6 | 1.2 | 0.4 | 1.6 | 1.0 | 0.8 | 1.5 |
| **GFRA1** | 1.2 | 0.8 | 4.2 | 1.1 | 0.7 | 2.2 | 0.9 | 0.1 | 1.2 | 1.1 | 0.6 | 1.3 |
| **GFRA2** | 0.9 | 0.6 | 4.5 | 0.9 | 0.7 | 1.5 | 0.9 | 0.5 | 1.4 | 1.0 | 0.4 | 1.6 |
| **GFRA3** | 0.8 | 0.4 | 2.9 | 1.2 | 0.4 | 1.5 | 1.1 | 0.4 | 1.7 | 1.0 | 0.3 | 2.1 |
| **NGF** | 1.1 | 0.7 | 2.7 | 1.8 | 1.1 | 7.5 | 1.1 | 0.7 | 1.9 | 1.0 | 0.8 | 1.5 |
| **NGFR** | 1.2 | 0.1 | 6.5 | 1.5 | 1.0 | 3.8 | 1.0 | 0.5 | 2.1 | 1.0 | 0.7 | 1.8 |
| **NGFRAP** | 0.9 | 0.4 | 1.2 | 1.0 | 0.7 | 1.5 | 1.0 | 0.7 | 1.3 | 1.0 | 0.8 | 1.2 |
| **NRG1** | 0.7 | 0.4 | 1.3 | 0.8 | 0.5 | 1.2 | 0.9 | 0.3 | 1.5 | 1.0 | 0.7 | 1.4 |
| **NRG2** | 1.2 | 0.6 | 1.8 | 1.3 | 0.9 | 2.7 | 0.9 | 0.6 | 2.4 | 0.9 | 0.7 | 1.8 |
| **NRG4** | 0.9 | 0.5 | 1.6 | 1.1 | 0.7 | 1.6 | 1.1 | 0.8 | 1.7 | 1.0 | 0.7 | 1.8 |
| **NTF3** | 1.1 | 0.5 | 2.4 | 1.3 | 0.9 | 2.3 | 1.1 | 0.8 | 1.8 | 1.0 | 0.5 | 1.4 |
| **NTF4** | 1.0 | 0.8 | 1.5 | 1.1 | 0.8 | 1.3 | 1.1 | 0.3 | 1.4 | 1.0 | 0.6 | 1.6 |
| **NTRK1** | 1.9 | 0.9 | 5.9 | 2.0 | 0.8 | 3.8 | 1.7 | 0.7 | 5.2 | 1.0 | 0.6 | 1.4 |
| **NTRK2** | 1.0 | 0.4 | 2.3 | 0.9 | 0.5 | 1.5 | 0.8 | 0.5 | 1.1 | 1.0 | 0.6 | 1.5 |
| **TGFA** | 1.2 | 0.9 | 1.9 | 1.2 | 1.0 | 2.2 | 1.1 | 0.7 | 1.6 | 1.0 | 0.7 | 1.3 |
| **NPY** | 0.5 | 0.2 | 2.7 | 1.3 | 0.5 | 3.5 | 0.9 | 0.4 | 2.6 | 1.2 | 0.5 | 1.7 |
| **NPYR1** | 0.8 | 0.3 | 2.9 | 1.0 | 0.8 | 1.7 | 1.0 | 0.5 | 1.5 | 1.0 | 0.4 | 1.6 |
| **MPZ** | 1.0 | 0.5 | 4.5 | 1.0 | 0.5 | 1.6 | 0.8 | 0.4 | 2.5 | 1.0 | 0.6 | 2.0 |
| **GFAP** | 2.1 | 1.2 | 7.5 | 1.6 | 0.4 | 3.3 | 1.2 | 0.4 | 2.6 | 1.0 | 0.5 | 1.7 |
| **FGF2** | 1.3 | 0.9 | 3.0 | 1.3 | 0.8 | 2.3 | 1.2 | 0.6 | 1.4 | 1.0 | 0.5 | 1.9 |
| **FGFR1** | 1.1 | 0.3 | 2.4 | 1.0 | 0.7 | 2.1 | 0.9 | 0.5 | 1.2 | 1.0 | 0.7 | 1.5 |
| **GDNF** | 1.5 | 0.4 | 3.5 | 1.4 | 0.8 | 2.1 | 1.0 | 0.6 | 2.3 | 1.0 | 0.7 | 1.7 |
| **ERBB2** | 1.2 | 0.7 | 3.1 | 1.2 | 0.9 | 2.7 | 1.1 | 0.7 | 2.4 | 1.0 | 0.7 | 1.23 |
| **ERBB3** | 1.1 | 0.5 | 3.0 | 1.1 | 0.9 | 1.8 | 1.1 | 0.7 | 1.6 | 1.0 | 0.8 | 1.3 |
| **ERBB4** | 1.2 | 0.7 | 1.8 | 1.4 | 0.8 | 2.8 | 1.4 | 0.9 | 1.9 | 1.0 | 0.4 | 1.6 |

Supplemental table 2.

Spearman's correlation Epidermal nerve fibre (ENF) count versus individual factors

| Variable | by Variable | Spearman ρ | Prob>\|ρ\| |
| --- | --- | --- | --- |
| ENF | CNTF | 0.1405 | 0.3355 |
| ENF | CNTFR | -0.1256 | 0.3900 |
| ENF | GALR1 | -0.2823 | 0.0494 |
| ENF | GALR2 | 0.0522 | 0.7335 |
| ENF | GFRA1 | -0.1851 | 0.2182 |
| ENF | GFRA2 | 0.1180 | 0.4242 |
| ENF | GFRA3 | -0.0175 | 0.9050 |
| ENF | NGF | -0.5704 | <.0001 |
| ENF | NGFR | -0.3350 | 0.0186 |
| ENF | NGFRAP | 0.1953 | 0.1787 |
| ENF | NRG1 | 0.2102 | 0.1561 |
| ENF | NRG2 | -0.2509 | 0.0820 |
| ENF | NRG4 | -0.1074 | 0.4625 |
| ENF | NTF3 | -0.2209 | 0.1272 |
| ENF | NTF4 | 0.0149 | 0.9201 |
| ENF | NTRK1 | -0.4032 | 0.0055* |
| ENF | NTRK2 | 0.0968 | 0.5081 |
| ENF | TGFA | -0.3147 | 0.0277* |
| ENF | NPY | 0.0268 | 0.8598 |
| ENF | NPYR1 | 0.0617 | 0.6738 |
| ENF | MPZ | 0.0266 | 0.8575 |
| ENF | GFAP | -0.3934 | 0.0057 |
| ENF | FGF2 | -0.2607 | 0.0704 |
| ENF | FGFR1 | -0.0859 | 0.5573 |
| ENF | GDNF | -0.2629 | 0.0711 |
| ENF | ERBB2 | -0.2223 | 0.1331 |
| ENF | ERBB3 | -0.1601 | 0.2719 |
| ENF | ERBB4 | -0.1123 | 0.4425 |
| ENF | BTM1 | -0.6330 | <.0001 |

Supplemental table 3.

Spearman's correlation Vibration perception threshold assessed by biothesiometer (BTM1) versus individual factors

| **Variable** | **by Variable** | **Spearman ρ** | **Prob>\|ρ\|** |
| --- | --- | --- | --- |
| BTM1 | CNTF | -0.0145 | 0.9240 |
| BTM1 | CNTFR | 0.1484 | 0.3249 |
| BTM1 | GALR1 | 0.3412 | 0.0203 |
| BTM1 | GALR2 | 0.2467 | 0.1152 |
| BTM1 | GFRA1 | 0.2590 | 0.0935 |
| BTM1 | GFRA2 | 0.1991 | 0.1897 |
| BTM1 | GFRA3 | 0.0150 | 0.9212 |
| BTM1 | NGF | 0.3416 | 0.0288 |
| BTM1 | NGFR | 0.3995 | 0.0066 |
| BTM1 | NGFRAP | -0.2040 | 0.1739 |
| BTM1 | NRG1 | -0.3253 | 0.0312 |
| BTM1 | NRG2 | 0.2276 | 0.1283 |
| BTM1 | NRG4 | -0.2660 | 0.0740 |
| BTM1 | NTF3 | 0.1828 | 0.2239 |
| BTM1 | NTF4 | -0.0009 | 0.9952 |
| BTM1 | NTRK1 | 0.3857 | 0.0106 |
| BTM1 | NTRK2 | 0.1597 | 0.2892 |
| BTM1 | TGFA | 0.1358 | 0.3683 |
| BTM1 | NPY | -0.0374 | 0.8118 |
| BTM1 | NPYR1 | 0.0458 | 0.7624 |
| BTM1 | MPZ | 0.0957 | 0.5316 |
| BTM1 | GFAP | 0.4912 | 0.0005 |
| BTM1 | FGF2 | 0.3758 | 0.0101 |
| BTM1 | FGFR1 | 0.2460 | 0.0994 |
| BTM1 | GDNF | 0.3839 | 0.0092 |
| BTM1 | ERBB2 | 0.0861 | 0.5784 |
| BTM1 | ERBB3 | 0.1473 | 0.3286 |
| BTM1 | ERBB4 | -0.0493 | 0.7448 |
